# Contrast echocardiography with a physiological contrast agent for alcohol septal ablation

**DOI:** 10.3389/fcvm.2025.1634818

**Published:** 2025-09-09

**Authors:** Erdogan Ilkay, Yasemin Saglam, Ersin Saricam, Bilge Duran Karaduman, Fehmi Kacmaz, Aysel Yakici, Cigdem Koca, Melike Polat, Zeynep Seyma Turinay, Mehmet Akif Erdol

**Affiliations:** ^1^ Department of Cardiology, Medicana International Ankara Hospital, Ankara, Türkiye; ^2^ Department of Cardiology, Guven Hospital, Ankara, Türkiye; ^3^ Cardiology Clinic, Saglik Bilimleri University, Ankara, Türkiye; ^4^ Faculty of Medicine, Üsküdar University, Istanbul, Türkiye; ^5^ Özel NEV Hospital, Sanliurfa, Türkiye; ^6^ Department of Cardiology, Sultangazi Educational and Research Hospital, Istanbul, Türkiye; ^7^ School of Medicine, Yeditepe University, Istanbul, Türkiye

**Keywords:** hypertrophic cardiomyopathy, alcohol septal ablation, myocardial contrast echocardiography, physiological contrast agent, myocardial opacification

## Abstract

**Purpose:**

Myocardial contrast echocardiography in alcohol septal ablation (ASA) is critical prior to alcohol injection into the target septal artery branch. However, current contrast agents are expensive, carry the risk of anaphylaxis reaction, and are widely unavailable. Against this background, in this study, we introduce a practical solution for the assessment of target septal arteries by using physiological, practical, and safer intracoronary injections.

**Methods:**

This study included 14 patients (8 females, 6 males), with symptomatic hypertrophic obstructive cardiomyopathy who underwent ASA between the years 2017 and 2025. Because of the unavailability and the high cost of contrast agents in our country, we used practical physiological agents, which comprised a cocktail fluid (agitated 5 mL of saline plus 0.5 mL blood of the patient). We injected ethanol (absolute alcohol 96%–99%) into the target perforatory artery using transesophageal echocardiography (TEE) in a step-by-step manner to observe a reduction in mitral regurgitation and QT prolongation at electrocardiographic monitorization (initially, 0.5 mL alcohol, then by increasing the dosage up to 3 mL).

**Results:**

We clearly obtained a good myocardial opacification of the interventricular basal septum border with our cocktail contrast agent. Furthermore, we used TEE in the ASA procedure, unlike other researchers who reported on this procedure. No arrhythmias and allergic reactions were recorded during the administration of the contrast agent. The mean dose of alcohol administered during ASA was 2.1 ± 0.7 mL. The procedural rate of success was highest (100%). We assessed the effectiveness of the treatment in terms of a reduction of the peak left ventricular outflow tract gradient and the disappearance of severe mitral regurgitation with a significantly systolic anterior motion.

**Conclusions:**

The use of a physiological cocktail fluid in TEE exemplifies the use of a practical, alternative myocardial contrast agent for alcohol septal ablation.

## 1 Introduction

Symptomatic patients with obstructive hypertrophic cardiomyopathy (oHCM) will require septal reduction therapy (SRT) if there is a failure of medical therapy (1, 2). Currently, both surgical septal myectomy and percutaneous alcohol septal ablation (ASA) are available as septal reduction therapy. The selection of an appropriate technique depends on structural characteristics, the experience of the surgical team in the given technique, and preferences of patients (3, 4).

ASA is defined as a selective injection of alcohol into the target septal perforator artery to create a localized septal scar. The application of this procedure leads to a reduction of the left ventricle outflow gradient, along with an alleviation of symptoms (5).

Identifying the target septal artery branch (TSAB) is the cornerstone of achieving the optimal benefit of septal reduction. Angiographic identification is not sufficient to detect the correct positioning of alcohol in the target myocardial area due to excessive anatomical variety and potential collateralization in the septal perforatory artery (6). Therefore, myocardial contrast echocardiography is critical prior to alcohol injection into the TSAB. The echocardiographic contrast agent is injected through the balloon catheter with simultaneous transthoracic echocardiography (TTE) and compared with the same views recorded at baseline. However, contrast agents such as Levovist and Gelafundin) are expensive, carry the risk of anaphylaxis reaction, and are widely unavailable (7). Hence, in this study we introduce a practical solution by using a simple, physiological, and safer contrast agent.

## 2 Methods

This observational cohort study included 14 patients with symptomatic oHCM (8 females, 6 males; mean age 52.6 ± 14.7 years). All patients underwent the ASA procedure between the years 2017 and 2025. All patients were duly informed about the procedure and written informed consent was obtained from each patient. This study was conducted in accordance with the principles of the Declaration of Helsinki. The study was approved by the Human Research Ethics Committee at Medicana International Ankara Hospital (BSH 2022/28-A).

A diagnosis of oHCM was made by two cardiologists based on typical clinical, electrocardiographic, echocardiographic, and cardiac magnetic resonance imaging characteristics. All patients had a baseline LV outflow gradient ≥30 mmHg at rest, and/or ≥50 mmHg were induced after provocation (1, 2).

All patients underwent comprehensive preprocedural evaluations, including TTE and transesophageal echocardiography (TEE). First, all of them were subjected to TTE for evaluating their interventricular septum and other cardiac functions. TEE was performed before the commencement of the main procedure for the purpose of detecting the diameter and shape of the interventricular septum and insufficiency of the mitral valve structure.

Significantly, systolic anterior motion (SAM) is a systolic anterior motion of the mitral valve with septal contact and the bending of mitral leaflets at the mid-portion.

The problem of mitral leaflet coaptation due to SAM causes severe mitral regurgitation. Successful ASA therapy reduces SAM and left ventricular outflow tract (LVOT) obstruction. Finally, severe mitral regurgitation associated with dynamic left ventricular outflow tract obstruction disappears.

The success criteria were defined as a reduction of the peak LVOT gradient to ≤30 mmHg at rest, eliminating severe mitral regurgitation with significant SAM (from 3 to 4 grade to ≤1 grade mitral regurgitation).

### 2.1 Percutaneous ASA technique

Femoral artery (7-Fr) access was obtained, and a temporary transvenous pacemaker was positioned through the right femoral vein or right jugular vein into the right ventricle apex. Then, baseline gradients were obtained, which included left ventricular and aortic pressures. Next, unfractionated heparin was administered at a dose of 100 IU/kg. A coronary angioplasty guide catheter through the femoral artery was inserted via the right femoral sheath and placed at the left coronary artery ostium (7 Fr guide catheter, extra backup). The percutaneous ASA procedure was performed under TEE guidance. We identified possible target septal branches in anterior–posterior cranial left coronary angiography or the right anterior oblique cranial position. Because the first septal artery branch commonly leads to left ventricular outflow tract obstruction, we considered it a target artery. An angioplasty floppy guidewire (0.014) was advanced into the TSAB, and a short (8–15 mm) compliant over-the-wire (OTW) balloon (1.5–2.5 mm, Boston Scientific, Maple Grove, USA) was inserted in the septal branch and inflated at nominal pressure (6–8 atm). This balloon was compatible with the injection of absolute alcohol and was positioned proximally after the takeoff of the septal branch to occlude it. The wire was removed afterward.

**Figure 1:**
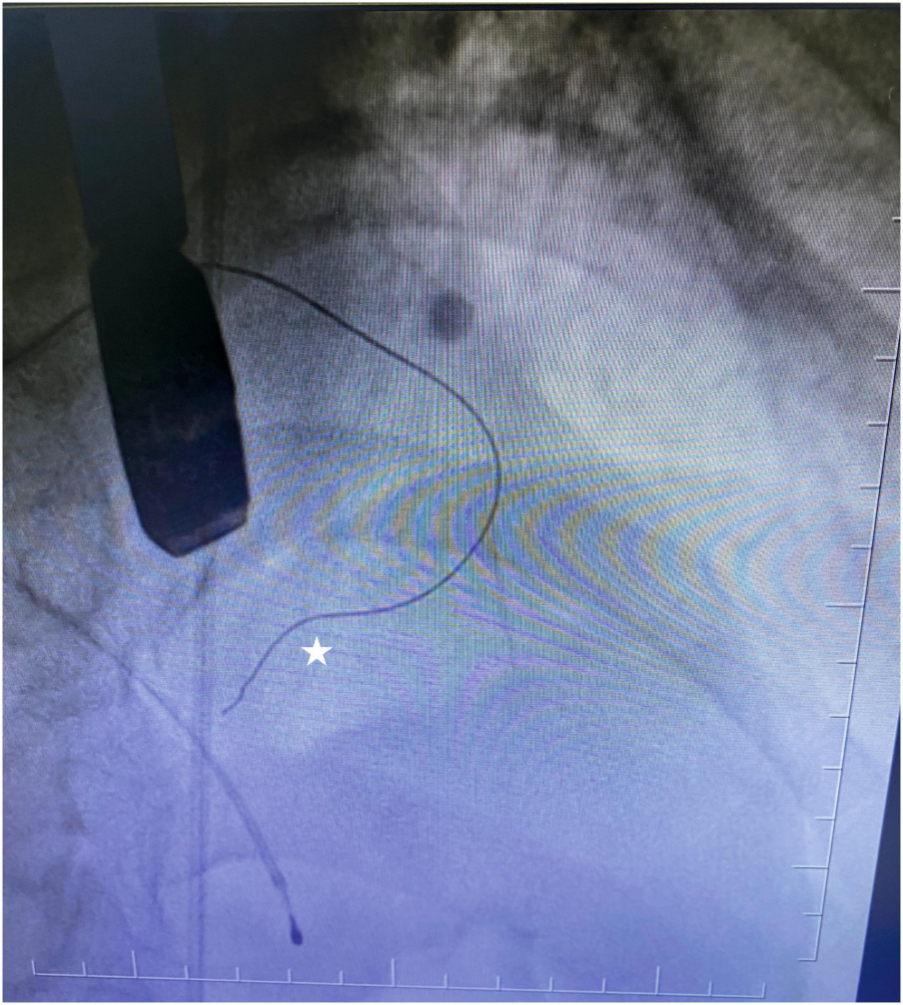
Coronary guidewire in the first septal branch.

To confirm the target myocardial area in the correct perforator artery, we used our contrast agent (agitated contrast substance) via TEE. The agitated contrast substance was injected through the balloon shaft (Figure 1).

To confirm the LV outflow tract obstruction–related septal artery, we reached the first septal artery by using the 0.014 guidewire. Then, the OTW balloon was advanced into the septal branch proximal segment and inflated at nominal pressure. The 0.014 wire was removed afterward. We administered 2 mL of our contrast agent (agitated contrast substance) through the OTW balloon. We observed the myocardial contrast area in the interventricular basal septum in TEE view. If it was the culprit septal artery, we injected alcohol into the target perforatory artery. If it was not, we repeated the same procedure step by step.

**Figure 2:**
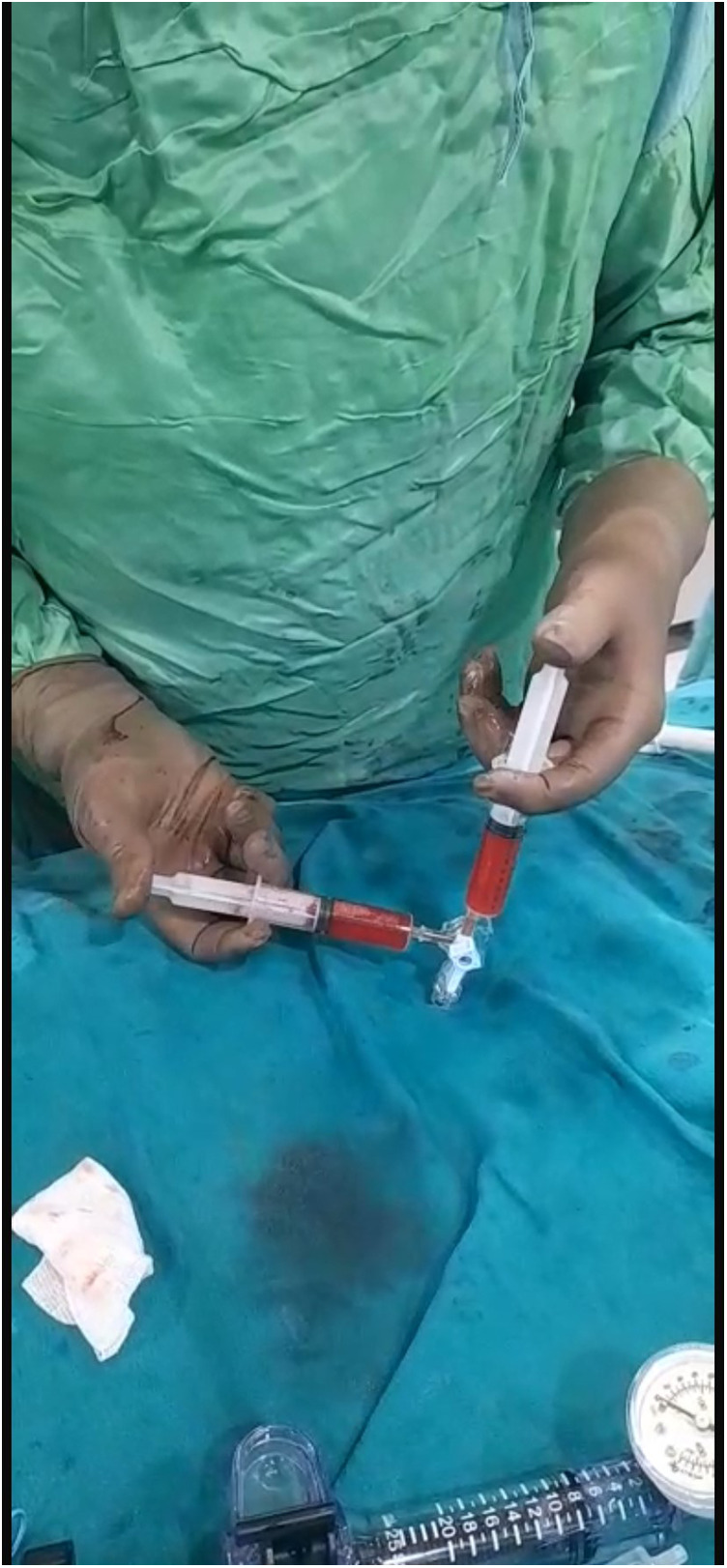
Cocktail fluid preparation.

**Figure 3:**
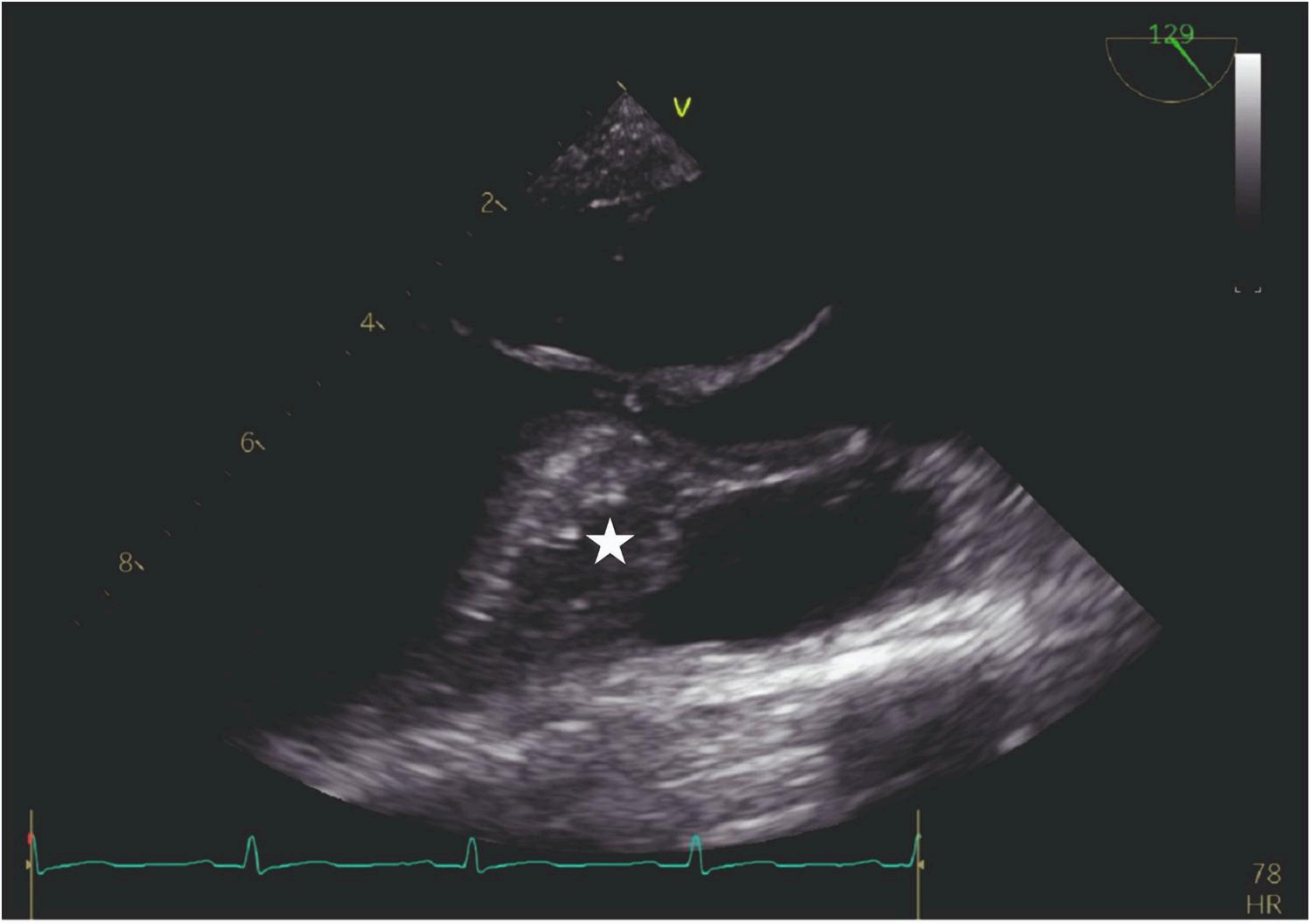
TEE view: the interventricular septum prior to the injection of cocktail fluid.

**Figure 4:**
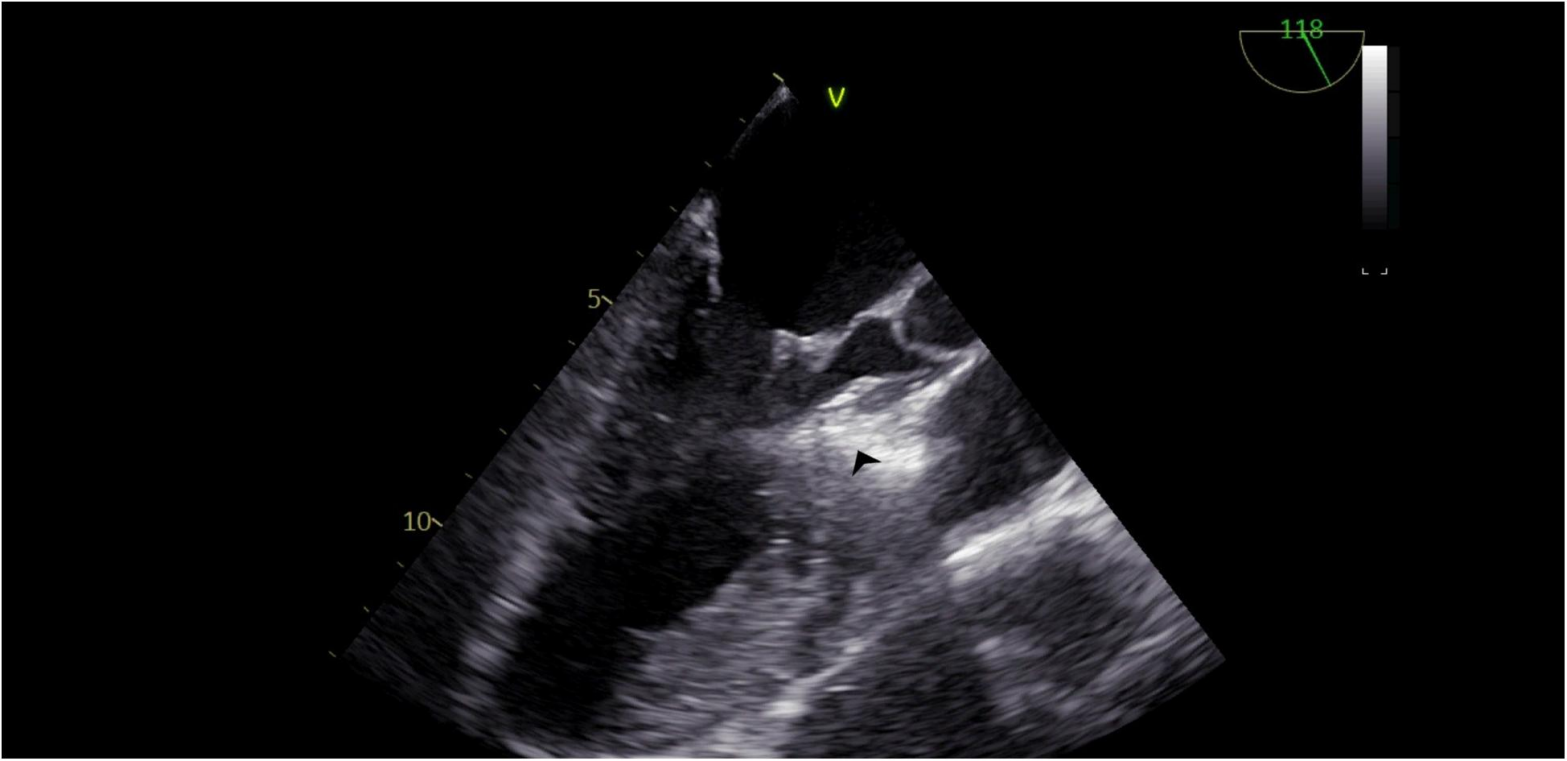
TEE view, following the injection of cocktail fluid through the central lumen of the over-the-balloon wire. Isolated brightness in the basal interventricular septum is clearly seen.

Our agitated contrast substance included cocktail fluid (agitated 5 mL of saline plus 0.5 mL blood of the patient, shaken in a three-way-stopcock (Figure 2). In TEE, the basal interventricular septum was compared with the same views recorded at baseline. Finally, we observed an enhancement of the basal septum (Figures 3, 4). Then, 1–2 mL of ethanol (absolute alcohol 96%–99%) was injected into the TSAB. We injected alcohol into the target perforatory artery in a step-by-step manner to observe a reduction in mitral regurgitation and QT prolongation at electrocardiographic monitorization (initially, 0.5 mL alcohol, then by increasing the dosage up to 3 mL). Two of the 14 patients had a single perforator artery with a double branch. In these patients, we performed superselective septal catheterization. Then, we administered contrast agents to both branches. Next, we found the correct branch feeding basal interventricular septum.

**Figure 5:**
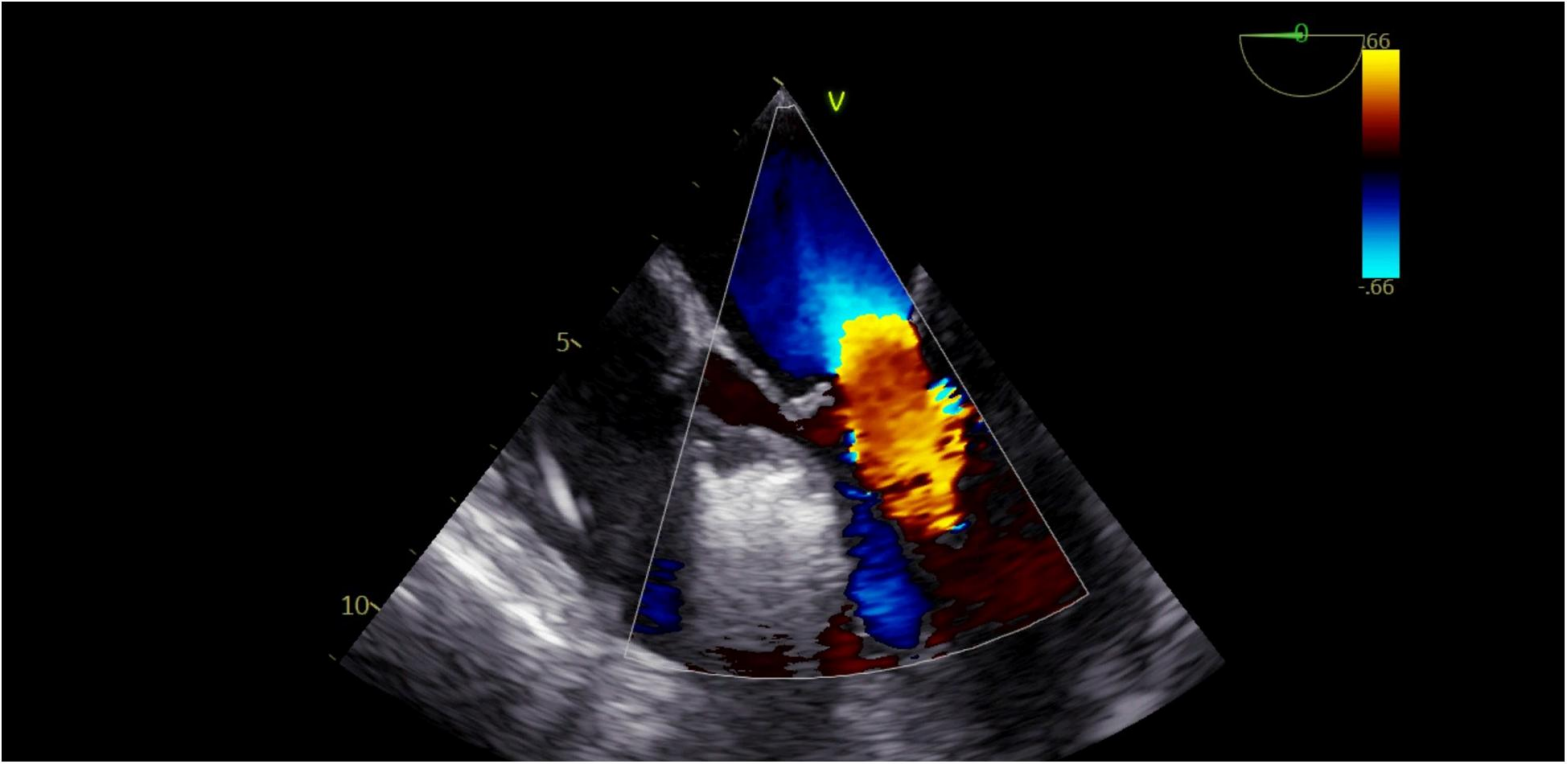
Disappearance of mitral regurgitation during the alcohol injection procedure.

We could clearly observe a reduction in mitral regurgitation during the procedure (Figure 5).

### 2.2 Statistical analysis

SPSS (Statistical Package for the Social Sciences, version17.0, SPSS Inc., Chicago, IL, USA) was used for statistical analyses. The results of the patients were reported using descriptive statistics (frequencies, means, and range).

## 3 Results

We included and analyzed a total of 14 patients with oHCM treated with ASA. In this entire cohort, demographic data, clinical histories, and risk factors were collected (8 females, 6 males) (Table 1).

**Table 1:** Clinical and echocardiographic characteristics of the study population at baseline and at the last checkup.

Patient features	
Female/male	**8/6** ^a^
Age, mean ± SD	52.6 ± 14.7
Interventricular septum thickness, mean cm	2.2
Initially LVOT gradient, mmHg	90.2 ± 12
After ASA gradient, mmHg	20.6 ± 5

a Patient number.

We clearly observed the related target area in TEE. We cared about the absence of the contrast in other myocardial areas for the safety of the technique in this procedure.

Furthermore, we did not encounter any complications related to its use in the form of backflow of bubbles to the left anterior descending artery. No arrhythmias were recorded during the administration of the contrast agent.

In addition, we monitored for possible complications such as pericardial effusion and ventricular septal defect.

After the procedure, all patients were put under observation in the coronary care unit for 24 h with a temporary pacemaker implanted in the body. When no episodes of AV block occurred, the periprocedural temporary pacemaker was removed. Because of its advantages of short hospitalization time and early recovery, ASA therapy has beneficial uses for oHCM. Such uses were seen in our patients as well.

The mean dose of alcohol administered during ASA was 2.1 ± 0.7 mL. The procedural success rate was highest (100%). We assessed the effectiveness of the treatment in terms of LVOT gradient decrease, residual SAM of the mitral valve, and mitral regurgitation reduction. Furthermore, we evaluated our patients according to the nature of their complications such as aortic insufficiency, complete heart block, and ventricular septal defect.

**Figure 6:**
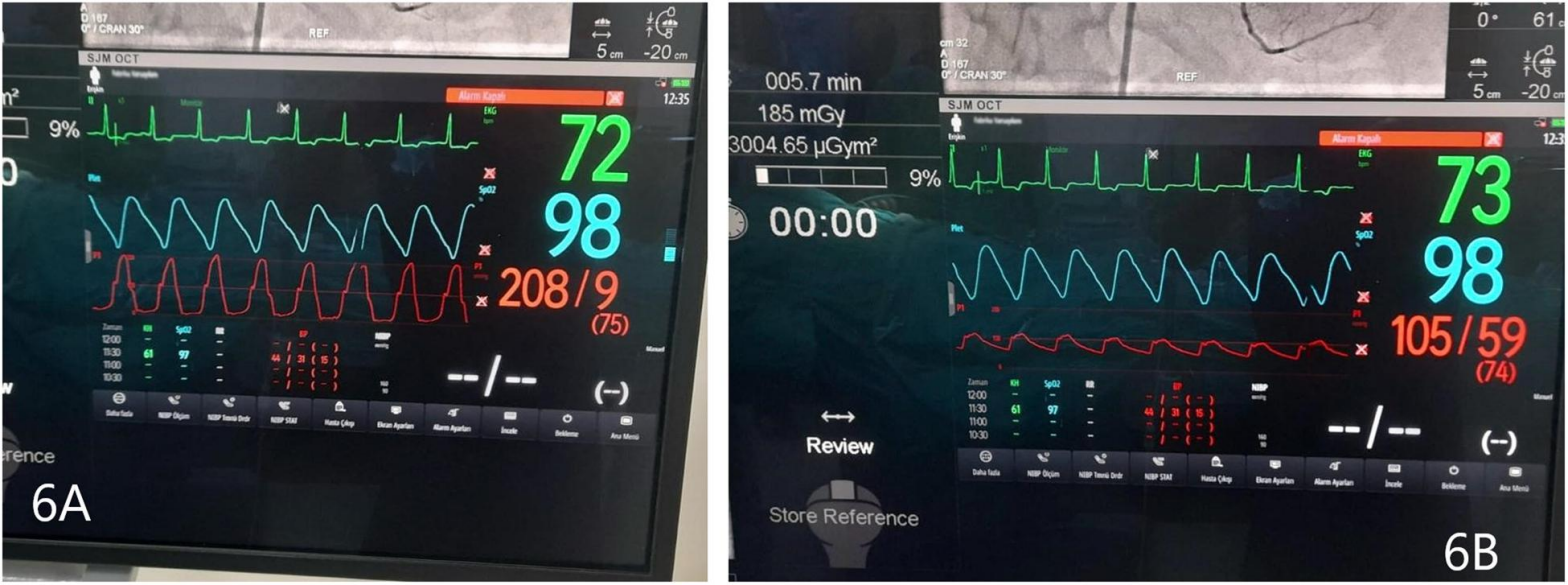
**(A)** Left ventricular pressure tracing, before ASA. **(B)** Aortic pressure, before ASA. Gradient 103 mmHg.

**Figure 7:**
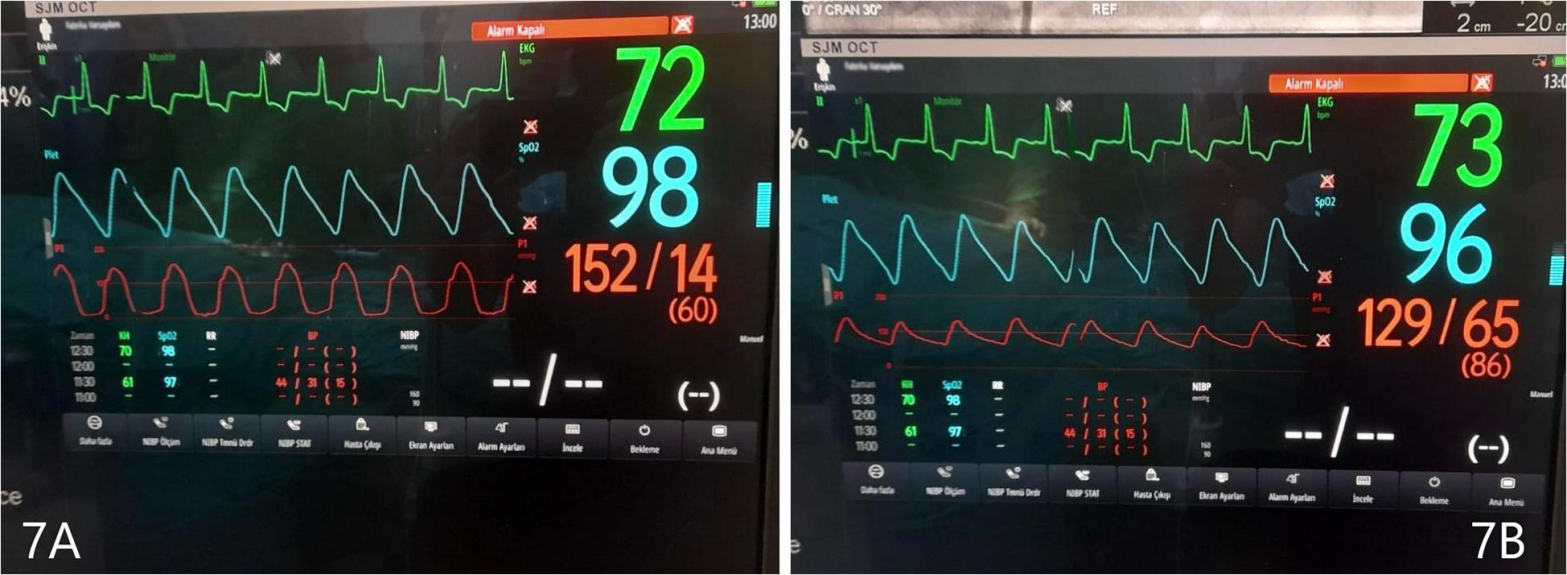
**(A)** Left ventricular pressure tracing, after ASA. **(B)** Aortic pressure, after ASA. Gradient 23 mmHg.

Before and after the ASA procedure, we recorded cardiac catheter findings (LVOT gradient, left ventricular pressure, and aortic pressure) (Figures 6A, B, 7A, B).

The procedural risk factors were arrhythmia (bradycardia or tachycardia), bleeding, prolonged chest pain, and pericardial fluid.

**Agitated saline-safety concerns**: We did not observe any allergic reactions and arrhythmias during target vessel identification through agitated contrast substances. We used 2 mL of agitated contrast substances for each procedure. This quantity was sufficient for visualization. In four patients, we did not identify the target septal vessel in the first evaluation. Therefore, there was again a need for target vessel identification; we applied an additional 2 mL of agitated contrast substance after 20 min.

**Short-term outcomes:** We assessed the factors of LVOT gradient decrease, residual SAM of the mitral valve, mitral regurgitation reduction, evidence of ventricular septal defect, and ventricular arrhythmias.

In the meantime, no patient needed any permanent pacemaker implantation.

## 4 Discussion

The most critical point of oHCM is LVOT obstruction, which is the most important mechanism responsible for symptoms such as heart failure and chest pain (8). Evidence for LVOT obstruction is associated with worse survival rates when compared with non-obstructive HCM (9, 10). According to the latest American and European Guidelines for HCM, patients with an LVOT gradient ≥50 mmHg either at rest or with provocation should be managed with SRT intervention in case of failed maximum tolerated medical therapy (1, 2). Currently, there are two main approaches for SRT in oHCM, namely surgical septal myectomy and percutaneous alcohol septal ablation (2).

The surgical therapy, septal myectomy, is an important procedure and allows gradient relief at any level within the ventricle (11–13). Surgical therapy is especially advantageous for treating coexisting anomalous papillary muscle, markedly elongated anterior mitral leaflet, intrinsic mitral valve disease, coexistent severe coronary artery disease, and valvular aortic stenosis (14–16).

ASA should be performed in reputed HCM centers if contraindications to surgery or high-risk cases are reported (1, 2). This procedure requires an asymmetric hypertrophy of the basal interventricular septum with a systolic anterior motion of the anterior mitral valve leaflet and more than 15 mm septal thickness at the region of obstruction; also, a suitable septal branch should be made available (17, 18).

Because of anatomical variety and potential collateralization, angiographic identification of the septal artery is not sufficient to detect the correct positioning of alcohol in the target myocardial area. Therefore, myocardial contrast echocardiography is necessary to test each target septal branch (17, 18). Several echocardiographic contrast agents were attempted for this intervention, including Levovist® (Bayer, Germany), agitated gelatin polysuccinate (Gelafundin®, BBraun, Melsungen, Germany), and SonoVue (gelatin polysuccinate) (6, 19, 20). However, these agents are unavailable in our country and in some other countries and also expensive (7, 21). Because of these constraints, we used practical physiological agents, which comprise cocktail fluid (agitated 5 mL of saline plus 0.5 mL blood of the patient). We clearly observed the interventricular basal septum border. We obtained good myocardial opacification with our cocktail contrast agent. Furthermore, we used TEE in the ASA procedure, unlike other researchers who reported on this procedure.

An ideal contrast agent must include the features of good contrast imaging of the target area, slow capillary runoff to confirm stable demarcation of the target area, rapid washout for safety, no allergic reaction, and no arrhythmogenicity (7). Our contrast agent encompassed all of these features.

## 5 Conclusion

The use of cocktail fluid in TEE exemplifies the use of a feasible alternative myocardial contrast agent for alcohol septal ablation.

## 6 Limitations

This study is not a comparative study with other contrast agents. The sample size of the study is small because it is a single-center study with limited data.

## Data Availability

The raw data supporting the conclusions of this article will be made available by the authors without undue reservation.
